# Assessing the Safety of Mesh Repair in Strangulated Groin Hernias: A Systematic Review and Meta-Analysis

**DOI:** 10.7759/cureus.70496

**Published:** 2024-09-30

**Authors:** Andrew C Ekwesianya, Abraham Ayantunde, Hussameldin M Nour

**Affiliations:** 1 Department of General and Colorectal Surgery, Southend University Hospital, Southend, GBR; 2 Department of General Surgery, Royal Lancaster Infirmary, Lancaster, GBR

**Keywords:** femoral, groin, hernia, infection, inguinal, mesh, recurrence, repair, seroma, strangulated

## Abstract

The use of mesh in emergency repair of complicated groin hernias has been a subject of discussion for decades. While it is now generally accepted that mesh could safely be used in incarcerated (irreducible) and obstructed hernias (without strangulation), with wound infection rates comparable to suture repairs, the use of mesh in strangulated hernias involving bowel resection is still controversial. The aim of this study, therefore, was to analyse the safety of mesh use in strangulated hernias with ischaemic bowel at the time of surgery. A literature search was carried out using relevant keywords. The study was conducted in accordance with the Preferred Reporting Items for Systematic Reviews and Meta-Analyses (PRISMA) 2020 framework anddata analysis was done using the Review Manager version 5.4 (The Cochrane Collaboration, Oxford, UK) meta-analysis software. Seven studies comprising 1,159 patients who had emergency surgery for strangulated groin hernias were analysed. A pooled random effect meta-analysis did not show any significant difference in the surgical site infection rate (odds ratio (OR) = 0.88, 95% confidence interval (CI) = 0.39-1.96, p = 0.75), seroma formation (OR = 3.39; 95% CI = 0.70-16.43; p = 0.13), and hernia recurrence (OR = 0.33; CI = 0.05-2.22; p = 0.26) between the two groups. The long-held concern that mesh could not be safely used in strangulated groin hernias has not been validated by the results obtained from this systematic review and meta-analysis. However, more randomised controlled trials in this clinical area would need to be carried out to further validate the results of this study.

## Introduction and background

Rationale for the review

Groin hernias are common conditions encountered in surgical outpatient clinics and emergency departments. The term groin hernia encompasses both inguinal and femoral hernias. The natural history of hernias generally involves progression from an asymptomatic reducible hernia to a symptomatic reducible hernia, an irreducible/incarcerated hernia, an obstructed hernia, and then to a strangulated hernia with gangrenous content. Though uncommon, if left unattended, the hernia could perforate through the skin and result in enterocutaneous fistula. Since the bowel is the most common content of a groin hernia, surgical repair of the hernia before it becomes complicated is usually the goal [1].

The repair technique introduced by Bassini using non-absorbable sutures revolutionised hernia suture repairs, resulting in a significant reduction in hernia recurrence rates [2]. However, the problem of tension inherent in the repair makes the recurrence rate still unacceptably high [3]. Bassini repair is now out of fashion in elective repair settings.

The advent of tension-free mesh repairs has further reduced the hernia recurrence rate, with minimal short- and long-term complications. In an analysis of pooled data from studies on hernia recurrence published over a 40-year period, Bassini and modified Bassini repairs were found to have a recurrence rate of 7.7% while Lichtenstein (tension-free) mesh repair had a recurrence rate of 0.5% [4]. Up to 95% of inguinal hernias are repaired with mesh in the National Health Service (NHS) England [5].

There is a variety of meshes based on the source (biological, synthetic or composite meshes), the pore size (macroporous, microporous), the weight (lightweight, heavyweight), and configurations [6]. Since mesh (especially synthetic mesh) is a foreign body, the most feared complication is surgical site infection, and this can occasionally result in mesh explantation [7].

The use of mesh in emergency repair of complicated groin hernias has been a subject of discussion for decades. While it is now generally accepted that mesh could safely be used in incarcerated (irreducible) and obstructed hernias (without bowel strangulation), with wound infection rates comparable to suture repairs [8,9], the use of mesh in strangulated hernias involving bowel resection is still controversial [10]. Most surgeons traditionally avoid the use of mesh (even if it is a biological mesh) in this setting.

Given that the risk of hernia recurrence is high in suture-based repair, especially when done in an emergency setting, the advantage of mesh repair in reducing hernia recurrence is too good to be ignored. This raises the question of how significant the risk of infection is when mesh repair is done in strangulated inguinal hernias.

There is a paucity of quality studies that have looked into the safety and efficacy of mesh repair in the setting of strangulated groin hernias, and many of the available studies are either retrospective or based on small sample sizes. The inherent concern that the use of mesh in this setting would inevitably result in infections has also prevented many surgeons from conducting well-structured research to statistically answer this clinical question.

Previous randomised controlled trials and meta-analyses that focused on the safety of mesh in emergency groin hernia repairs incorporated data from incarcerated, obstructed (without bowel ischaemia or strangulation) and strangulated (with bowel ischaemia) hernias [9,11]. Even a meta-analysis that was done on 'strangulated inguinal hernia' also included data from incarcerated, non-strangulated hernias in the analysis [12]. As such, there is no dedicated meta-analysis that focuses only on strangulated hernias and the safety of mesh use in this clinical setting.

Objectives

The aim of this study, therefore, was to analyse the safety of mesh use in only a subset of emergency groin hernias - strangulated hernias - with ischaemic bowel at the time of surgery, commonly requiring bowel resection. This study carried out a systematic literature search on already available retrospective and prospective studies on this subject and then performed a meta-analysis on the pooled data to determine the safety of mesh repair in strangulated groin hernias. The primary outcome measure was surgical site infection while the secondary endpoints were seroma formation and recurrence rate.

## Review

Methodology

Eligibility Criteria

The inclusion criteria for this study include inguinal and femoral hernias presenting as an emergency; prospective and retrospective studies; open and laparoscopic mesh repair; mesh and suture repair; the presence of strangulation at the time of hernia repair, confirmed intraoperatively or evidenced by bowel resection.

Studies excluded from the analysis include those with a combination of data from incarcerated (irreducible), obstructed and strangulated hernias in which separate data for strangulated hernias was not reported; studies that incorporated strangulated hernias of other abdominal sites (e.g., incisional hernia, umbilical hernias); and case reports.

Search Strategy and Data Collection Process

The literature search was carried out on SCOPUS, PubMed, Embase and the COCHRANE Library databases, on 10 December 2023. The period searched was from 1980 to 2023. The search terms used were ‘mesh’, ‘repair’, ‘strangulated’, ‘groin’, ‘inguinal’, ‘femoral’, ‘hernia’, ‘recurrence’, ‘seroma’, ‘infection’, ‘emergency’. The Boolean operators (AND, OR, NOT) were used to widen or narrow the search parameters as appropriate. An additional manual search of the references in identified studies and meta-analyses on this subject was also conducted, and extra data was extracted for analysis. The literature review followed the PICO (Population/Patient, Intervention, Comparison and Outcome) framework [13].

Outcome Measures

The outcomes of interest assessed in this study were surgical site infection, seroma formation and hernia recurrence. In addition, other data parameters extracted included the study type, design, patient demographics, technique of mesh and suture repairs, and type of mesh used.

Synthesis Methods

The study was conducted in accordance with the Preferred Reporting Items for Systematic Reviews and Meta-Analysis (PRISMA) 2020 framework [14]. Data analysis was carried out using the Review Manager version 5.4 (The Cochrane Collaboration, Oxford, UK) meta-analysis software [15,16]. Odds ratio (OR) with 95% confidence interval (CI) was used to calculate the statistical difference in outcome between the intervention and control groups. Heterogeneity was assessed using the Chi-squared test, with statistical significance set at p-value < 0.05. The I^2^ test was used to quantify the degree of heterogeneity, with <30% indicating mild heterogeneity, 30-60% indicating moderate heterogeneity, and >60% indicating severe heterogeneity [17].

Results

Study Selection

**Figure 1:**
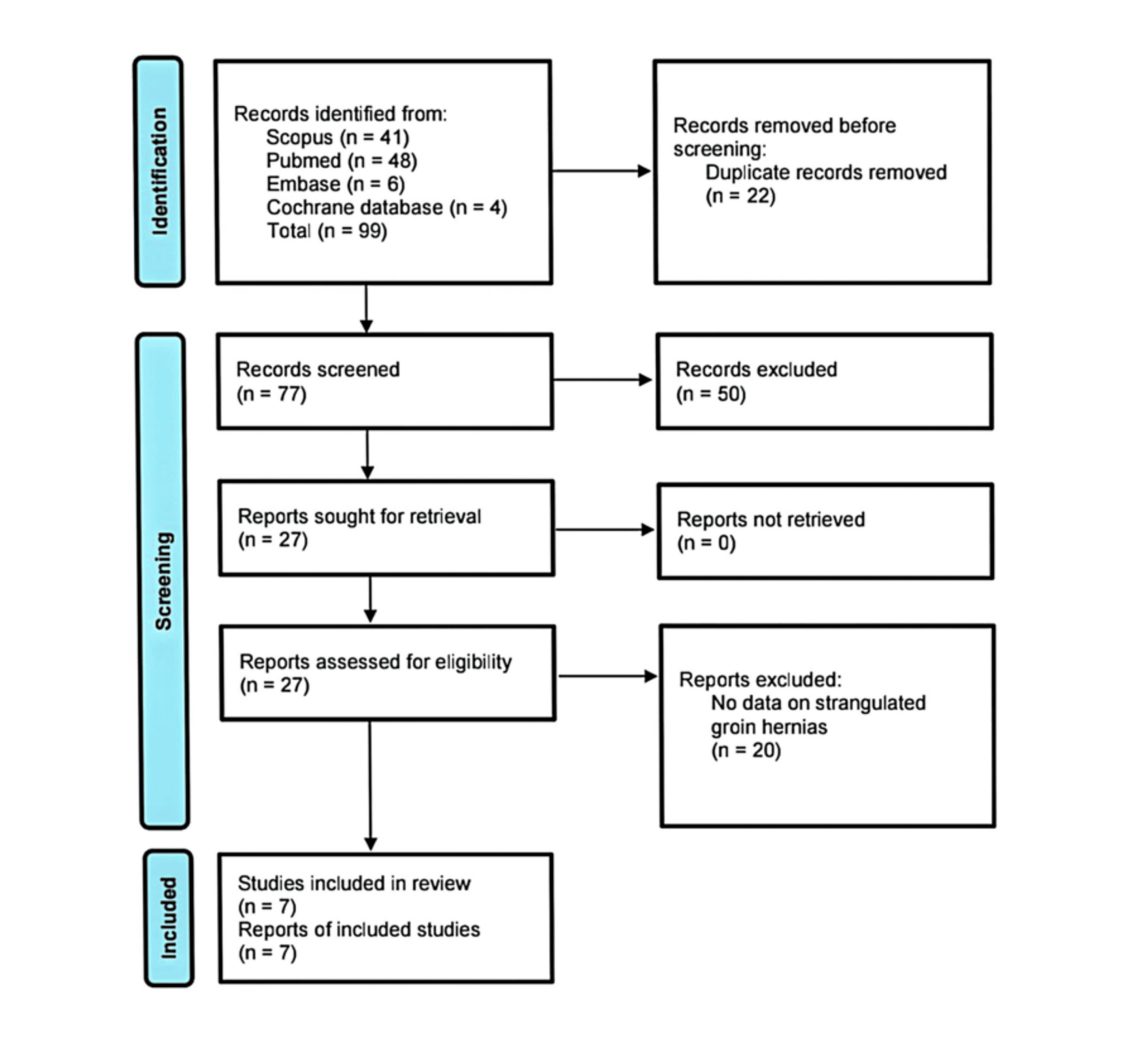
PRISMA 2020 flow diagram for updated systematic reviews that include searches of databases and registers PRISMA: Preferred Reporting Items for Systematic Reviews and Meta-Analyses

A literature search of the databases identified 99 studies on emergency repair of groin hernias, 22 of which were duplicate records. The titles and abstracts were analysed against the inclusion criteria, resulting in the exclusion of 50 articles. Following further scrutiny of the methodology and outcome measures of the remaining 27 articles, only seven of them were deemed eligible and included in the systematic review, as shown in the PRISMA flowchart in Figure 1.

Study Characteristics

The seven studies included in the meta-analysis were published between 2005 and 2022. Two were randomised controlled trials [18,19], one was a prospective non-randomised study [20], while the other four were retrospective studies [21-24]. Two studies [19,23] included both inguinal and femoral hernias, while the others were on inguinal hernias alone. While the other six studies compared tension-free mesh repair and suture repair, Bessa et al. [20] compared the outcome of emergency mesh (Lichtenstein) repair versus elective mesh (Lichtenstein) repair in a randomised controlled fashion. The characteristics of these studies are outlined in Table 1.

**Table 1:** Baseline characteristics of studies included in the meta-analysis

Study	Year	Country	Study Type	Hernia Type	Study Design	Mean Age (Years)	Mesh Type	Mean Follow-Up
Papaziogas et al. [21]	2005	Greece	Retrospective	Inguinal	Tension-free repair vs modified Bassini	69.8 ± 7.4	Polypropylene	9 ± 4.2 years
Wysocki et al. [22]	2006	Poland	Retrospective	Inguinal	Lichtenstein vs Bassini/Shouldice	-	Polypropylene	35.8 months
Bessa et al. [20]	2007	Egypt	Prospective	Inguinal	Emergency Lichtenstein vs Elective Lichtenstein	-	Polypropylene	20 months
Elsebae et al. [18]	2008	Egypt	Prospective	Inguinal	Lichtenstein vs Bassini	34.6	Polypropylene	22 ± 6 months
Ueda et al. [23]	2012	Japan	Retrospective	Inguinal and femoral	Lichtenstein vs Bassini	79.5	Polypropylene	20 months
Duan et al. [19]	2018	China	Prospective	Inguinal and femoral	Lichtenstein vs Bassini	54	Polypropylene	-
Sakamoto et al. [24]	2022	Japan	Retrospective	Inguinal	Mesh vs non-mesh repair	-	-	-

Results of Individual Studies

A summary of the data on the outcome measures obtained from the individual studies is presented in Table 2. A total of 478 patients were included in the intervention group while 681 patients were in the control group. All the studies analysed the outcome of surgical site infection postoperatively, whereas only four studies [18,20-22] reported on postoperative seroma occurrence. Similarly, hernia recurrence was analysed in only four of the studies, with a follow-up range of 20 months to nine years and a median follow-up of 45 months [18,20,21,23].

**Table 2:** Results of primary outcomes in both intervention and control groups

Study	Total		Infection		Seroma		Recurrence	
	Mesh Repair	Suture Repair	Mesh Repair	Suture Repair	Mesh Repair	Suture Repair	Mesh Repair	Suture Repair
Papaziogas et al. 2005 [21]	33	42	2	4	2	0	1	2
Wysocki et al. 2006 [22]	56	21	2	3	2	0	-	-
Bessa et al. 2007 [20]	25	25	0	0	1	0	0	0
Elsebae et al. 2008 [18]	27	27	1	3	1	0	0	3
Ueda et al. 2012 [23]	10	17	2	3	-	-	0	0
Duan et al. 2018 [19]	104	104	32	16	-	-	-	-
Sakamoto et al. 2022 [24]	223	445	6	15	-	-	-	-
Total	478	681	45	44	6	0	1	5

Results of Pooled Analysis

**Figure 2:**
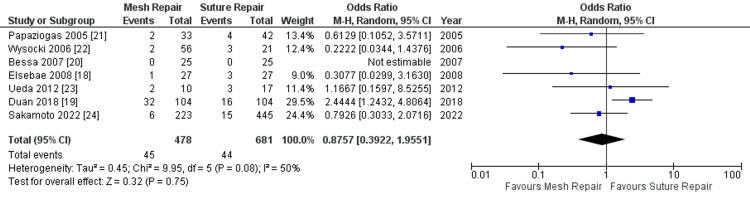
Forest plot of comparison: mesh repair versus suture repair; outcome: surgical site infection

Surgical site infection: Surgical site infection, which was assessed in all the studies, occurred in 45 (9.41%) of patients in the intervention group and in 44 (6.46%) of patients in the control group. A pooled random effect meta-analysis did not show any significant difference in the surgical site infection rate between the groups, as shown in Figure 2. The OR for the overall effect was 0.88 at 95% CI: 0.39-1.96, with a p-value of 0.75. There was moderate heterogeneity (I^2^ = 50%).

**Figure 3:**
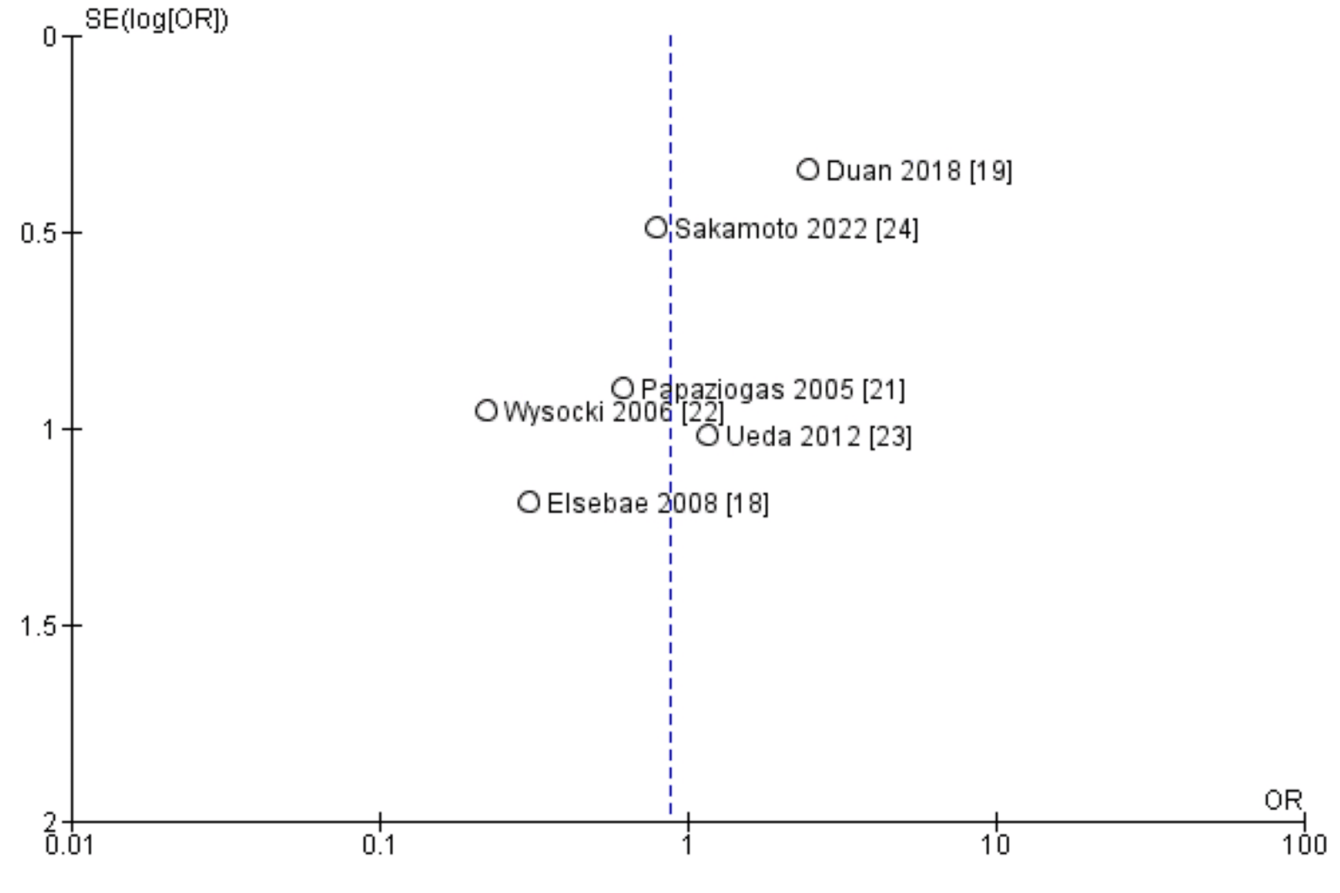
Funnel plot of comparison: mesh repair versus suture repair; outcome: surgical site infection

The heterogeneity is attributable to the results of the study by Duan et al. [19] in which surgical site infection was significantly less in the control group than in the intervention group. This is further confirmed in the Funnel plot (Figure 3) in which this study by Duan appears as an outlier in the plot.

**Figure 4:**
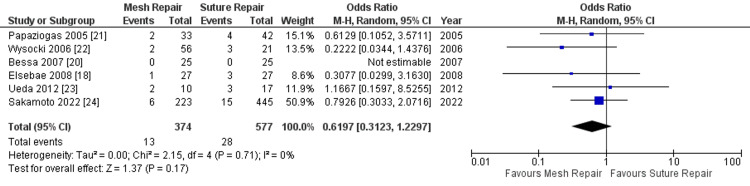
Forest plot of comparison (after removal of outlier study): mesh repair versus suture repair; outcome: surgical site infection

When the outlier study is excluded, a repeat meta-analysis of the remaining six studies still shows there is no significant difference between the two groups (OR = 0.62, 95% CI: 0.31-1.23, p = 0.17), but this time with a heterogeneity (I^2^) of 0%, as shown in Figure 4.

**Figure 5:**
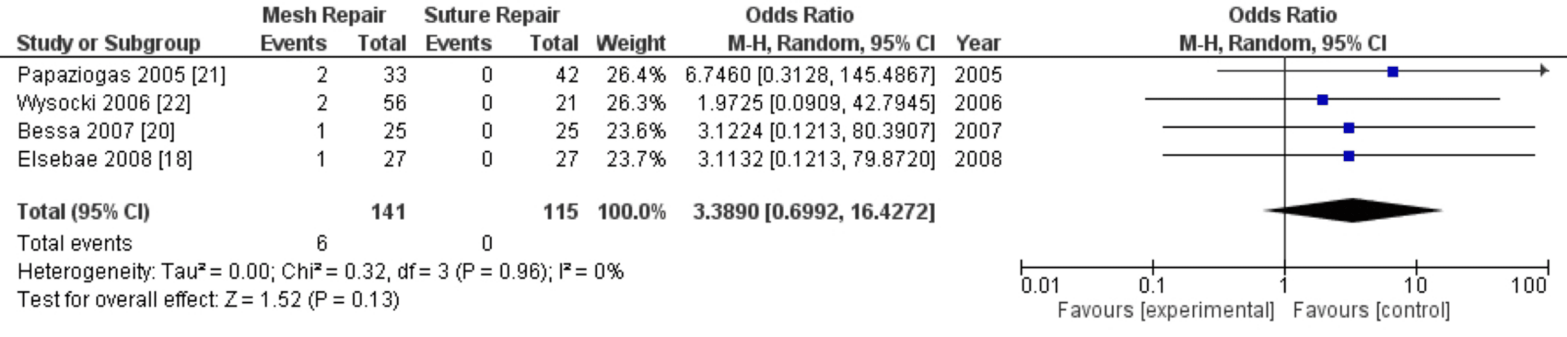
Forest plot of comparison: mesh repair versus suture repair; outcome: seroma formation

Seroma formation: From the four studies that reported postoperative seroma formation, this complication occurred in six of the 478 patients in the intervention group (1.26%) and none in the control group (0%). However, a meta-analysis of the pooled data did not show this difference to be statistically significant (OR = 3.39; 95% CI: 0.70-16.43; p = 0.13), as illustrated in Figure 5. There was low heterogeneity (I^2^ = 0%).

**Figure 6:**
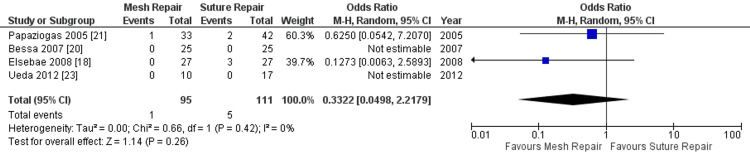
Forest plot of comparison: mesh repair versus suture repair; outcome: hernia recurrence

Hernia recurrence: Hernia recurrence, reported in only four of the studies, occurred in one patient in the intervention group (0.21%) and in five patients in the control group (0.73%). As shown in Figure 6, the random effect meta-analysis did not show any statistically significant difference in the hernia recurrence rate between the two groups (OR = 0.33; 95% CI: 0.05-2.22; p = 0.26; I^2^ = 0%).

Discussion

This is a systematic review and meta-analysis of four retrospective and three prospective studies comprising 1,159 patients who had emergency surgery for strangulated groin (inguinal and femoral) hernias. A total of 478 patients had emergency mesh repair of their strangulated groin hernia while 681 patients were in the control group. The pooled meta-analysis did not show any statistically significant difference in surgical site infection between mesh and suture-based repairs, contrary to long-held concern and belief that the use of mesh is not safe in this clinical setting.

This finding is similar to the results of a previous meta-analysis involving 1241 patients who had emergency groin hernia repair, 626 of whom had hernia repair with mesh and 615 had non-mesh repair. Even though the study looked at all emergency groin hernia repairs (both strangulated and non-strangulated hernias), there was no statistical certainty that the 90-day surgical site infection rate was higher following mesh repair than suture-based repair (relative risk (RR) 1.00, 95% CI: 0.15-6.64) [25].

Our study also found that the rate of seroma formation in the early postoperative period is not significantly different irrespective of the type of hernia repair (mesh or suture-based). This outcome is also similar to the results of a meta-analysis carried out by Marcolin and colleagues on incarcerated and strangulated groin hernias, in which no significant difference was found in seroma formation between patients who had mesh repair and those who had suture repair of their hernias (OR 0.80; 95% CI: 0.49-1.32; p = 0.39) [9]. Seroma formation is, however, also affected by the size of the hernia sac and the surgical approach for the hernia repair.

Reduced hernia recurrence rate is one of the significant advantages that the use of mesh has over suture repair in hernia surgery, and this underlies the rationale for consideration of mesh in emergency settings, including in strangulated hernias. In the meta-analysis by Marcolin et al., the use of mesh significantly reduced hernia recurrence when compared with primary suture repair (OR 0.36; 95% CI: 0.19-0.67; p = 0.001). This finding remained consistent and unchanged even after sensitivity analysis [9]. Similarly, a meta-analysis by Hentati and colleagues reported that mesh repair had a significantly lower recurrence rate over primary suture repair in the emergency setting (OR 0.20; 95% CI: 0.05-0.78, p = 0.02) [12].

Our study, however, did not find any statistically significant difference between the patients who had mesh repair and those who had primary suture repair of their strangulated hernia (OR = 0.33; 95% CI: 0.05-2.22; p = 0.26). Tastaldi and colleagues, in their retrospective analysis of 257 patients who underwent emergency groin hernia surgeries and were followed up for 43 months, also did not find any difference in recurrence rate between mesh repair and suture repair groups [8].

The outcome in the recurrence rate identified in our study might have been influenced by the sample size and the duration of follow-up. Only four studies comprising a total of 206 patients (95 in the mesh repair group and 111 in the suture repair group) analysed hernia recurrence. Out of these, only one study had a mean follow-up of nine years [18]; others followed up their patients for only a period of 20-22 months [20,21,23]. The calculated median follow-up was 45 months. This short period of follow-up was not long enough to identify most of the recurrences, as some might have recurred after the follow-up was discontinued.

Limitations

This systematic review incorporated a total of seven studies, comprising four retrospective and three prospective studies. Given that retrospective studies provide lower-quality scientific evidence, the overall quality of the review would be affected by the quality of the individual studies. In addition, the small sample size in some of the studies used in the review would limit the applicability of the results to the general population. There is a real concern among surgeons about mesh infection and having to explant an infected mesh. This might affect not just the number and scope of studies, but also the study design and introduce recruitment bias.

## Conclusions

The use of mesh in the repair of incarcerated (irreducible) and obstructed groin hernias has been well-established by randomised controlled trials and meta-analyses. Such, however, is not the case for strangulated hernias in which the contents have become ischaemic, sometimes requiring bowel resection. The primary objective of this review was to assess the safety of mesh repair in this subset of emergency groin hernias in which the contents have become strangulated. The long-held concern that mesh could not be safely used in strangulated groin hernias has not been validated by the results obtained from this systematic review and meta-analysis. However, more randomised controlled trials in this clinical area need to be carried out to further validate the results of this study.
